# Rethinking attitudes to student clinical supervision and patient care: a change management success story

**DOI:** 10.1186/1472-6920-14-182

**Published:** 2014-08-30

**Authors:** Maree O’Keefe, Victoria Wade, Sue McAllister, Ieva Stupans, Jennifer Miller, Teresa Burgess, Amanda LeCouteur, Linda Starr

**Affiliations:** Faculty of Health Sciences, The University of Adelaide, Adelaide, Australia; Faculty of Health Sciences, Flinders University, Adelaide, Australia; School of Sciences and Technology, University of New England, Armidale, Australia

## Abstract

**Background:**

The aim of this project was to explore the process of change in a busy community dental clinic following a team development intervention designed to improve the management of student supervision during clinical placements.

**Methods:**

An action research model was used. Seven members of a community dental clinic team (three dentists, two dental therapists, one dental assistant and the clinic manager), together with the university clinical placement supervisor participated in the team development intervention. The intervention consisted of two profiling activities and associated workshops spread six months apart. These activities focused on individual work preferences and overall team performance with the aim of improving the functioning of the clinic as a learning environment for dental students. Evaluation data consisted of 20 participant interviews, fourteen hours of workplace observation and six sets of field notes. Following initial thematic analysis, project outcomes were re-analysed using activity theory and expansive learning as a theoretical framework.

**Results:**

At project commencement students were not well integrated into the day-to-day clinic functioning. Staff expressed a general view that greater attention to student supervision would compromise patient care. Following the intervention greater clinical team cohesion and workflow changes delivered efficiencies in practice, enhanced relationships among team members, and more positive attitudes towards students. The physical layout of the clinic and clinical workloads were changed to achieve greater involvement of all team members in supporting student learning. Unexpectedly, these changes also improved clinic functioning and increased the number of student placements available.

**Conclusions:**

In navigating the sequential stages of the expansive learning cycle, the clinical team ultimately redefined the ‘object’ of their activity and crossed previously impervious boundaries between healthcare delivery and student supervision with benefits to all parties.

## Background

Clinical placements are an integral component of student education in health professions such as medicine, nursing, dentistry and allied health. They offer students opportunities to develop and refine their clinical skills under expert supervision, as well as developing teamwork skills in an authentic, and often interprofessional, setting. Day-to-day clinical practice is highly variable and unpredictable especially when patient load is high. To manage this complexity, responsibility for student supervision during clinical placements often rests with a designated, discipline-specific clinician who acts as a link between the clinical team and the student. Whilst sensible from a management perspective, such an approach can inhibit student interaction with the wider clinical team and potentially limit the range of authentic learning opportunities.

Simplification of the learning environment runs counter to the diversity and complexity that lies at the heart of clinical healthcare [1]. It can also be argued that, to better reflect clinical practice, management of student supervision in the clinical environment should be a shared, rather than an individual responsibility. Activity theory, and the associated model of expansive learning provides a useful theoretical lens through which organisational changes in health services can be examined and understood [2–4]. Activity theory considers the various activities people engage in to achieve particular outcomes (the ‘object’ of the activity) as well as the ways in which these objects are achieved (tools). ‘Activity systems’ comprise interactions between the people involved in the activity, their personal objectives, the communities in which they participate, the tools used to achieve these objectives, the ‘rules’ in play, and the organisation of work tasks [1]. Within a single organisation, multiple activity systems may co-exist, for example activity systems around patient care may exists alongside activity systems around student clinical supervision within a health service.

Achieving effective change in an organisation may require a transformation in understanding or ‘expansion’ of the object of the activity. As the object is reconceived with an expanded understanding of the inherent purpose of the activity, so too are the associated activities and, in some cases, members ‘cross boundaries’ between previously separate activity systems to realign their objectives with those of the new system [5]. ‘Boundary objects’ may facilitate movement between activity systems. These are objects such as patient case records that are used in multiple activity systems, but that can have different functions within each individual activity system [6, 7].

The aim of this project was to explore the process of change in a busy community dental clinic following a team development intervention designed to improve the management of student supervision during clinical placements.

## Methods

The project was conducted at a community dental clinic in South Australia in 2008–2009. An action research model underpinned the study design to enable a rigorous and reflective evaluation. The Human Research Ethics Committee of the University of Adelaide approved the project and formal consent was obtained from all participants. The qualitative component adheres to the RATS guidelines for reporting qualitative studies [8].

At the time of the project, and as part of their clinic final year of study, dental students from the University of Adelaide School of Dentistry provided treatment to patients under the supervision of qualified dentists. The clinic was hosting rotations of two new students each fortnight and there was general but nonspecific concern among clinic team members that the clinic was not functioning optimally as a student learning environment. When approached by the project team the participating clinic staff saw the project an opportunity to gain a better understanding of their current arrangements to support student learning and to explore alternative approaches.

Seven clinic staff (three dentists, two dental therapists, one dental assistant, one manager) and the university placement coordinator agreed to participate in the development activities and associated evaluation that were conducted over a nine month period. One dental assistant and one dental therapist declined participation. The intervention consisted of two profiling activities and associated workshops spread six months apart. These activities focused on individual work preferences and overall team performance with the aim of improving the functioning of the clinic as a learning environment for dental students. Specific details of the intervention are described elsewhere [9]. ‘Problem’ areas with student clinical placements were identified and participants developed an improvement plan to address these problems. This plan was implemented and refined in the six months between the two workshops. Members of the project team visited the clinic on two occasions during this time to discuss progress with participants in an informal setting.

Evaluation data were collected through participant interviews and workplace observation. SM interviewed participants on two occasions: at the beginning of the project (staff baseline) and in the week prior to the second workshop six months later (staff follow-up). Two dental students who were placed at the clinic when the project began were also interviewed (student baseline). Approximately eight weeks after the second workshop, an independent evaluator conducted a focus group interview attended by five clinic members and one student (staff/student exit). The other two clinic staff and one student were either unavailable or declined participation. Interviews were semi-structured with questions informed by the goals of the project (see ‘Participant interview questions’ section). In the baseline interview, participant perceptions of team roles, responsibilities and general team functioning around student learning were sought. In the follow-up interview approximately six months later, the extent to which the team’s engagement with the development activity had been associated with changes in team functioning was explored. In the exit interview, participant perceptions, expectations and experiences during the project were discussed. The interviews were digitally recorded with de-identified transcripts produced.

### Participant interview questions

*Baseline Interview (staff)*

How does your team manage student learning?

What do you think students learn?

How do they learn those things?

What works well when you have students placed with you?

What do you think could be done better when students are placed at your service?

*Baseline Interview (student)*

What is it like for you being part of this clinical team?

How do members of the clinical team support your learning?

What suggestions do you have to improve the learning experience for students?

*Follow-up Interview (after 6 months; staff)*

What has it been like for you participating in this project?

Was it useful having your TMP profile identified and explained?

What do you think it’s been like for the team to participate in this project?

Have you noticed any changes in the way the team manages student learning?

Was it useful to identify team goals?

How do you know when students’ learning is going well?

Have you changed anything you do to support students’ learning?

What part of the project so far has been most/least useful for you or the team?

*Exit focus group (staff/student)*

What one or two things stand out for you personally?

Did the early phases of the project help you to know what to expect from your participation?

Has the dental service developed as a place for students to learn?

What will enable you to maintain and further develop students’ learning?

To what extent has the Project been an enabler of change?

Detailed field observations within the clinic prior to each workshop were also recorded by SM with a particular focus on student activity and interaction with clinic staff. In addition, SM, MOK and IS recorded field notes at the workshops regarding participant interaction and engagement. Examples of activities observed included logging the staff with whom the students were interacting, the length, initiator, and purpose of the interaction (teaching/supervising, operational, social, other). In addition, notes were made in relation to the structuring of the learning environment including the physical placement of students in relation to other members of the clinical team. The amount of time patients were kept waiting while a student sought assistance was also noted. During the workshops field notes recorded the level of engagement with, and contribution to, discussion and activities by individual clinical team members. The duration of the first observation period was eight hours and the second was six hours. These time periods represented the time students were in the clinic on the observation days.

Interview, observation and field-note data were entered into NVivo software v8 [10]. Thematic analysis was conducted regarding participant perceptions of team functioning in relation to student supervision at baseline and on changes that followed the intervention [11]. VW undertook initial open coding of data into categories that were subsequently verified by MOK, SM and ALC. Recurring themes were identified following review and discussion by all authors. The appropriateness of the identified themes in capturing all relevant data, and the interrelationships between themes, was tested by iterative cycles of project team discussion and comparison of identified themes against the original transcript data. Triangulated comparisons were made between interview data, observational data and field notes to strengthen the validity of the findings. Where inconsistencies were identified in either the themes identified by individual researchers, or between interview and observational data, these findings were discussed among the project team members with reference back to the original transcript data, until a consensus view emerged as to the appropriate recording of such a finding. Where differences between team members occurred further discussion was undertaken until consensus was achieved.

Following the thematic analysis of the evaluation data, the project outcomes were re-analysed using activity theory and, in particular, expansive learning as a theoretical framework [5]. This framework conceives successful change as one that arises from a sequential transformation of understanding among key individuals as to the purpose (object) of their work activities. The model comprises seven sequential stages: *Questioning* - to capture the current need; *Analysis -* to understand the tensions at play; *Modelling the new solution -* a key stage to enable an innovative break though in thinking; *Examining and testing the new model –* where ideas are debated; *Implementing the new model; Reflecting on the process –* where short comings and/or resistance are identified; *Consolidating and generalising the new practice –* where the team can move to a new starting point.

The research team comprised a range of academic health professionals with backgrounds in medicine, oral health, speech pathology, pharmacy, nursing and psychology. None of the project team was directly involved in training dental students or providing health care within the participating clinic. The researchers met frequently to debrief their observations and to explore any particular disciplinary perspectives they may have brought to their interpretation of interview or observational data. The multidisciplinary nature of the research team enabled a great richness of perspectives to be brought to the process of analysis. In addition team members had an appreciation of the complexity of the clinical environment through their individual professional experience. This included familiarity with the dynamic nature of the relationship between student, patient and supervising clinician within each unique clinical encounter. The ways in which the presence of an observer might have influenced clinic activities were also monitored. This was done through liaison with the practice manager and dental therapists to monitor appointment schedules for aberrations, noting references (visual, verbal or otherwise to the observer), or any other disruptions to normal work flow. No evidence of a significant change to routine clinic activities arising from the observation could be identified. The research team was welcomed within the clinic for formal activities such as workshops.

## Results

**Figure 1:**
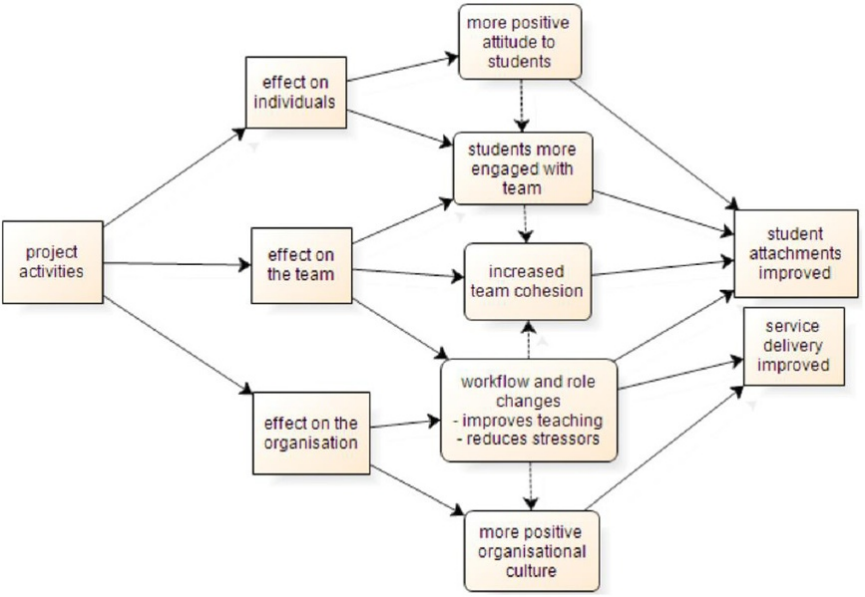
**Project Outcomes on Dental Clinic Functioning.** Brings together, in schematic form, the outcomes of the initial thematic analysis.

Twenty transcripts, fourteen hours of workplace observation and six sets of workshop field notes were generated. The initial thematic analysis produced a model of project outcomes at the individual, team, and organisational levels, as shown in Figure 1. Participants and senior management deemed the project to have been a success in achieving changes in student learning and concurrent improvements in workplace culture and service delivery. Staff reported more positive attitudes towards students and a greater sense of inclusion in the clinical team. Workflow and staff role changes implemented as a result of the project were reported to have delivered improved clinical service delivery and a more positive work culture [9].

To understand the processes that had underpinned such a successful outcome, the progress of the clinical team through the life of the project was mapped to the expansive learning cycle [5]. This process commenced with a clarification of the ‘problems’ that were understood to exist within the clinic.

### Pre-existing concerns with student clinical placements were identified (Questioning)

In the baseline staff interviews participants characterised student supervision as a burden on the clinic, and expressed concern about their ability to concurrently provide patient care. Different levels of individual student proficiency were seen as a complicating factor that precluded a standardised approach by the team to supervision. Moreover, both dentists and other team members regarded student supervision as the responsibility of the dentists, and of one dentist in particular. At this point in the process, participants could identify key challenges but were unable to identify and/or implement effective strategies to improve management of student supervision during placements.

Students were treating patients in two rooms that were separate to the rest of the clinic. The dentists responsible for providing clinical supervision for these students were also treating patients in a third room. The physical separation of the individual students and the supervising dentist meant that the students had to leave their own room and patient to speak with the dentist whenever they needed to ask for assistance. The fact that the supervising dentist was also treating a patient concurrently meant that the student and the student’s patient had to wait until the dentist had completed their own consultation. Even though the dental assistants were working alongside each of the students, the workplace observations provided evidence that the students interacted predominantly with the supervising dentists, only directing simple procedural questions to the dental assistants, even though the latter were present in the room.

### The first workshop clarified the challenges facing the team (Analysis)

During the first workshop, inherent tensions associated with the need to provide patient care and student supervision simultaneously were explicitly identified and discussed. Difficulties with workflow were highlighted including the additional time taken in student consultations:

*[Before the project began] the patients were sitting around in the chair waiting a long time before they saw the student …, it was just a huge inconvenience sometimes for a patient to see a student. (Staff exit)*

*It’s very awkward if you have to wait for 20 minutes for the dentist to come [and check your work] … I’m not comfortable and neither is the patient.* (*Student exit)*

When prompted, participants expressed concern at their own lack of effective teamwork and collaboration to manage student supervision. Participants agreed they were not all comfortable with leaving the full teaching responsibility to the dentist, and that there was potential for some sharing of student supervision. The dental assistants, for example, reported instances in which they could have made specific suggestions directly to the student but felt it was not their role to do so for example, when a patient became anxious.

### The first workshop supported the development of possible solutions and provided the catalyst for implementation (Modeling the new solution)

The workshop created an opportunity for dialogue about valuing the existing diversity of skills and interests among team members. Participants became animated when they looked in detail at their own and their co-workers’ preferences across different teaching roles, leading to a ‘break through’ moment; participants found that they shared a desire for using this diversity to improve workplace interactions around student supervision.

*I think also throughout the whole course of this program it [the workshop] emphasized the uniqueness of everyone and how we all worked together in this team. (Staff exit)*

These insights led to the team reflecting on its current performance in managing student supervision. Discussion then shifted from a ‘wish list’ of changes and aspirations to an actual plan for change.

*Within this study everyone listens and we deal with even how to make what you’re suggesting into a reality as much as possible. (Staff 6 follow-up)*

### Following the first workshop changes were proposed and tested (Examining and testing the new model)

Over the six months between workshops team members trialed different approaches to managing student clinical supervision. These included modifying staff roles to give greater responsibility to team members other than dentists, systematically collecting student feedback, changing the patient appointment booking system, and consulting students regarding optimal appointment timings to assist their learning prior to each placement commencing. Additionally, the team changed the physical layout of the clinic so that students were moved out of individual side rooms and grouped together in an open area visible to all members of the team.

### The second workshop supported the success of the new model and affirmed its value to participants (Implementing the new model)

A new model for managing student supervision was in place by the time of the second workshop. As part of this new approach, whenever students were present the clinic cancelled patient bookings for one of the dentists so that each student could receive increased supervision and feedback. With additional supervision available, the clinic took more students per rotation and the waiting time for patients to be seen was reduced because more students were consulting and treating patients. Greater numbers of students in the clinic also led to the employment of an additional dental assistant. There was general agreement that the new model was an improvement, not only in terms of student supervision but also in staff perceptions of patient satisfaction.

*It’s worked well for the community too, cause it gives people someone else to see … I’ve not had one [patient] that’s come out and said, I don’t want to see a student again. Whereas we used to get that on a fairly – not regular – but I used to get it a bit. … Having a tutor [dentist] that’s focused only on what the students are doing to patients makes it seem more important for the patients. (Staff exit)*

The reconfiguration of the clinic’s physical layout to facilitate student supervision also resulted in the clinic functioning more efficiently for other staff. Students did not need to wait to obtain assistance from the dentist who was always close by.

*I think the [dentists] are more relaxed at the moment, because they don’t have the patient load, and being interrupted and all that sort of thing. It’s not as stressful as it was last year, with running around trying to find the [dentist] and having to wait, wait, wait. Running behind, running late for lunch. … Running late at night. That’s not happening now. The assistants are able to manage them [students] better … You can see what they’re up to and you can see whether they’re struggling or not. (Staff 1 follow-up)*

Rather that representing competing priorities for participants’ time, students were recognised as contributing to high-quality patient outcomes.

*On a day to day basis [now] the students aren’t sort of talked about as pains and [that we] get held up because they’re slow, or whatever. I think there is a greater commitment to have them on board and accept them. (Staff 4 follow-up)*

One aspect of the project that was repeatedly commented on was the systematic collection and dissemination of all student feedback, both positive and negative, to team members. Previously, only negative feedback was reported, and often in an ad hoc manner.

*The feedback forms were something that was really good … you think why wasn’t this thought of before … It seemed so obvious. … When we have our little meetings and [name] will get the forms out and it’s good you feel [a] pat on the back, that’s good. (Staff 2 follow-up)*

### Exit interviews gave an opportunity for debriefing (Reflecting on the process)

Participants reported a change in workplace culture with more secure and positive relationships developing between individuals. Participants were uniformly positive about the improved performance of the team in their discussions and began to see that their team had a collective responsibility to provide clinical service and high quality student clinical placements.

*This year’s been very happy, hasn’t it? The whole clinic, it’s developed and changed. (Staff exit)*

*This is the fourth or fifth group of students [since the project began], every one of them has come at the end [of the placement] and come up to me and said, ‘Thank you so much. This was so much more than we had expected. (Staff 6 follow-up)*

Participants reported that the team had collectively developed its role in learning and teaching, with dental assistants more actively involved in providing feedback to students during procedures. Field-note observations confirmed these reports.

*I’ve thought about it [role in student learning] because before I didn’t really give it very much thought at all. …. It wasn’t very defined previously what do we do, and do we have a role in it and just how can we sort of help or improve things. Where now certainly that’s made it clearer I think. (Staff 2 follow-up)*

### Consolidating and generalising the new practice (onto a new starting point)

Supporting student learning became an important goal for the team and students were more clearly understood to add value to service rather than just being a drain on resources.

*The students are now like part of the team where before, initially, I think they were just seen more as an inconvenience, or not really part of us and what we do, where now I think they are very much [a part of our team]. (Staff exit)*

*The work environment … had a more social atmosphere than on the previous observation. Students had their own space but were rarely on their own in the work area, unlike the previous observation and the tutor was almost continuously present at a distance…. students were active contributors, and the goals set were influenced by the students’ input. (Follow-up observation)*

By the end of the project there was increased student input into treatment decisions, broader team input into student feedback and the implementation of a new system for patient bookings to accommodate identified student learning needs and preferences. Field observations supported the dental team’s assessment that they were now acting more as a team rather than as a group of individuals, and that a number of points of tension were no longer problematic. Student feedback was positive at the conclusion of the project.

*I feel confident here, not isolated in an enclosed cubicle on my own where I can’t easily ask for help. (Student exit)*

In addition to reporting actual changes, participants stated their intention and motivation to continue positive changes in the clinic. The team was assisted in this intention by strong support from senior management within the dental service, some of whom subsequently became champions of the project and the achievements of the clinic staff.

## Discussion

Organisational changes introduced into the clinic were associated with an improved clinical supervision environment for students, and improved patient service delivery. Participating clinical team members developed more positive attitudes towards students, and a greater appreciation of the value of students contributing actively to the planning of their learning experiences. Greater team cohesion, a more positive attitude towards students, and workflow changes delivered efficiencies in practice and enhanced relationships among team members. An increased range of options for students to contribute to patient care also became available.

Through the course of this project, the team developed a new understanding of their role. They transformed and expanded the object of their activity from a predominantly clinical service focus to one where the management of the student clinical placement also assumed significance. Clinical team members moved from a primary focus on clinical activities to a combined service/supervision activity system. This change or ‘boundary crossing’ was not confined to a single team member, but was embraced by all members of the clinical team. The patient appointment booking system functioned as a boundary object in this context [6, 7]. In addition to organising clinical activities, an additional function for this appointment template was created in relation to managing student clinical placement supervision. The appointment booking system then provided a stable framework for clinical activities and student supervision activities to occur together [12].

When the project began the existing activity system of the clinic was based around the object of delivering high quality patient care. In theory, participants understood the importance of training future dentists, but in practice, team members were highly focused on clinical tasks [13]. Student supervision was viewed as an activity undertaken by dentists, not the team as a whole, and as a disruption or impediment to clinical activity. Focusing the team’s attention on student clinical supervision in addition to the provision of clinical care was associated with a transformation of the object of the team’s activity, a key feature of expansive learning [2, 5]. These changes were most obviously manifest in the physical re-organisation of the clinic, and the greater involvement of all members of the clinical team in facilitating and supporting student learning [14]. The resulting employee initiated improvements in clinic efficiency, while maintaining patient care, made it likely that these changes would be sustained beyond the life of the project [15].

This project was a small study conducted in a single dental clinic thus limiting the extent to which specific findings might be generalisable. However, the challenges encountered by team members responsible for supervising and supporting student learning, and the themes expressed by them in interviews on the topic, are common across healthcare discipline clinical placements [16]. It should be noted that the dental clinic was a relatively self-contained health service with the capacity to reorganise the ways in which team members delivered dental services and organised student learning. Prior to their participation in the project, many members of the clinical team already met regularly as a cohesive group to discuss clinical matters and were thus well placed to embrace expansive learning and change [17]. Further work is now underway to test the value of this approach to understanding the process of change in clinical learning environments for other healthcare professionals working within more complex organisational structures [18].

Although engagement with this clinical team occurred over an extended period, the actual intervention time was relatively short, being two one-day workshops and two goal-review meetings. Spreading project activities over a relatively long period of time enabled the engagement and support of senior management as part of the process which proved beneficial in the context of this project in securing structural changes to service delivery.

Whilst only two students were placed at the clinic at any one time, the changes made at this clinic as a result of the project had an impact on the experiences of approximately 60 students each subsequent academic year. It should be noted also that this project was focused primarily on the clinic staff and although students were interviewed, there was no assessment of the impact of the project on their learning outcomes. There already exits significant literature on student learning in clinical placements, whereas this intervention focused on a relatively neglected area, that of health service staff and the organisational aspects of student placements. The student experience was recognised as an important consideration and the dental clinic continued to monitor student evaluations and feedback of their experiences throughout the project. Similarly patient satisfaction was not formally evaluated to support staff perceptions and anecdotal reports of improvements associated with the new model of service.

## Conclusions

In navigating the sequential stages of the expansive learning cycle, the clinical team ultimately reconceived the ‘object’ of their activity. In redefining the nature of their activity system, team members crossed previously impervious boundaries between healthcare delivery and student supervision with benefits to all parties.
